# Extracellular Adenosine Generation in the Regulation of Pro-Inflammatory Responses and Pathogen Colonization

**DOI:** 10.3390/biom5020775

**Published:** 2015-05-05

**Authors:** M. Samiul Alam, Matthew G. Costales, Christopher Cavanaugh, Kristina Williams

**Affiliations:** Immunobiology Branch, Office of Applied Research and Safety Assessment, Center for Food Safety and Applied Nutrition, US Food and Drug Administration, Laurel, MD 20708, USA; E-Mails: matthew.costales@gmail.com (M.G.C.); Christopher.Cavanaugh@fda.hhs.gov (C.C.); Kristina.Williams@fda.hhs.gov (K.W.)

**Keywords:** adenosine, CD39, CD73, adenosine receptors, immune response, lymphocytes, macrophage, bacterial-persistence, inflammation, cytokine

## Abstract

Adenosine, an immunomodulatory biomolecule, is produced by the ecto-enzymes CD39 (nucleoside triphosphate dephosphorylase) and CD73 (ecto-5'-nucleotidase) by dephosphorylation of extracellular ATP. CD73 is expressed by many cell types during injury, infection and during steady-state conditions. Besides host cells, many bacteria also have CD39-CD73-like machinery, which helps the pathogen subvert the host inflammatory response. The major function for adenosine is anti-inflammatory, and most recent research has focused on adenosine’s control of inflammatory mechanisms underlying various autoimmune diseases (e.g., colitis, arthritis). Although adenosine generated through CD73 provides a feedback to control tissue damage mediated by a host immune response, it can also contribute to immunosuppression. Thus, inflammation can be a double-edged sword: it may harm the host but eventually helps by killing the invading pathogen. The role of adenosine in dampening inflammation has been an area of active research, but the relevance of the CD39/CD73-axis and adenosine receptor signaling in host defense against infection has received less attention. Here, we review our recent knowledge regarding CD73 expression during murine Salmonellosis and *Helicobacter*-induced gastric infection and its role in disease pathogenesis and bacterial persistence. We also explored a possible role for the CD73/adenosine pathway in regulating innate host defense function during infection.

## 1. Introduction

The immune response is a highly regulated and interconnected cellular system that functions to maintain and restore the steady-state condition in the host. This mission is accomplished by guarding organs for indication of microbial invasion or tissue injury triggering protective inflammatory and corrective responses following infection or injury [1,2]. The balance between immune activation and suppression is intricately regulated, allowing optimal host response against infection, while simultaneously limiting collateral immune-mediated damage to the host tissues. One biomolecule that has been implicated in the control of host response to infection and tissue injury is adenosine. Adenosine is produced from ATP (adenosine-5'-triphosphate) catabolized by enzymes CD39 (nucleoside triphosphate dephosphorylase) and CD73 (ecto-5'-nucleotidase) which are expressed by myriads of cells and tissues. Any alteration of this catabolic machinery can change the outcome of many pathophysiological events, such as autoimmune diseases, atherosclerosis, ischemia-reperfusion injury, cancer and infections [1]. Also, the production and expression adenosine/CD73 can be regulated by the inflammatory milieu (cytokines) [3] and can impact both innate and adaptive immune responses during infection. In this review, we briefly discuss the recent knowledge and advancement in the host expression of CD39/CD73 and adenosine receptor (AR) signaling during both intracellular and extracellular Gram-negative bacterial infections and their role in the disease pathogenesis.

## 2. Adenosine Generated by CD39/CD73 Expression and Its Mechanism of Action

Adenosine is a purine nucleoside that accumulates in inflamed or hypoxic tissues largely due to the action of nucleoside triphosphate dephosphorylase mediating the dephosphorylation of ATP to ADP (adenosine-diphosphate) and then to 5'-adenosine mono-phosphate (AMP), which is the substrate for CD73/ecto-5'-nucleotidase that catalyzes the terminal reaction to convert 5'-AMP to adenosine [4,5]. The numerous responses mediated by adenosine act *via* four G-protein-coupled P1 purinergic cell surface receptors (GPCR): A_1_, A_2A_, A_2B_ and A_3_. Adenosine receptors function through the inhibition or stimulation of adenylyl-cyclase to decrease or increase intracellular cyclic adenosine monophosphate (cAMP) levels: A_1_ and A_3_ receptors activate an inhibitory regulative G-protein (Gi)-mediated decrease in cAMP levels, while stimulative regulative G-protein (Gs) receptors of the A2 family increase cAMP concentrations [1,5,6]. Adenosine might also enter cells by passive and active transporter channels and exert its effects inside the cells independently of receptor engagement [3,7]. Extracellular adenosine signaling functions to prevent excessive inflammation by suppressing proinflammatory cytokines, inhibiting leukocyte entry into tissues through down-regulation of adhesion molecules and chemokines, and triggering the production of anti-inflammatory cytokines such as IL-10 [8,9,10].

During inflammation, multiple cell types release extracellular ATP (eATP) either by cell lysis or non-lytic mechanisms [1]. The first step in the metabolism of nucleotides (ATP) is accomplished by CD39, which dephosphorylates extracellular ATP to AMP. CD39 expression has been studied in several cell types of mouse and human immune systems, including neutrophils, monocytes, B cells, natural killer (NK) cells, NK T cells and is present on the surface of helper T cells [11,12,13,14,15]. Pulte *et al.* described CD39 expression and activity in human leukocytes during disease states [13]. The expression of CD39 can also be regulated by proinflammatory cytokines, oxidative stress, and hypoxia [1,16,17] through transcription factors Sp1 [17], Stat3, and zinc finger protein growth factor independent-1 (Gfi-1) transcription factor [18].

Ecto-5'-nucleotidase/CD73 [EC 3.1.3.5] is the rate-limiting enzyme in adenosine production. The second and terminal step in the metabolism of purines is accomplished by CD73, which dephosphorylates extracellular AMP to produce adenosine. CD73 is a ubiquitously expressed protein in many immune cells and tissues [19]. CD73 is also expressed in diverse mouse cells [20]. In both human and mouse immune systems, CD73 is expressed in subsets of T cells, myeloid cells, bone marrow stromal cells, thymic epithelial cells and human B cells [21]. Numerous reports showed that CD4^+^CD25^+^Foxp3^+^ regulatory T cells (Treg) express CD39 and CD73 and that their presence enhances Treg cell function through the production of adenosine [22,23,24]. Reports suggest that the expression and function of this enzyme are generally upregulated under hypoxia [25,26], as well as by the presence of several pro-inflammatory cytokines, including tumor growth factor (TGF)-β, interferons (IFNs), tumor necrosis factor (TNF)-α, interleukin (IL)-1β and prostaglandin E2 [27,28]. Recent work by Niemela *et al.* [29] showed that IFN-α induces a time- and dose-dependent long-term up-regulation of CD73 in endothelial cells, but not in lymphocytes, both at the protein and RNA (ribonucleic acid) levels. Moreover, CD73-mediated production of adenosine is increased after IFN-α treatment of endothelial cells, resulting in a decrease in the permeability of these cells. So, various cytokines, including IFN-α, can be a relevant *in vivo* regulator of CD73 expression in the endothelial-leukocyte microenvironment during infection/inflammation. In activated hepatic stellate cells, up-regulation of CD73 transcripts are reported to occur *via* specific Sp1 and Smad promoter elements [30]. Recent evidence suggests that the intestinal microenvironment is also a preferential site for the generation of adenosine [31]. We and others have shown that in both mice and human gastric tissues, CD73 is abundantly expressed and activation can modulate its expression [21,31,32,33,34]. Furthermore, CD73 expression and downstream adenosine signaling are critical in the compensatory responses to tissue ischemia [9,35,36].

Besides host cells, many pathogens (e.g., *Escherichia coli*, *Staphylococcus aureus*, *Streptococcus agalactiae*, *Toxoplasma gondii*, and *Trichomonas vaginalis*) are armed with CD39/CD73-like machinery, that can aid pathogen colonization and dissemination [37,38,39,40]. Further, *Staphylococcus aureus* adapts to exploit immunosuppressive pathways to increase its own survival [39]. Reports suggest that pathogens produce adenosine from AMP via adenosine synthase A (AdsA), an extracellular ectonucleotidase expressed on the surface of the cell wall of bacterium which allows evasion of host immune surveillance [39]. Other studies demonstrate the existence of bacterial ectotriphosphate diphophohydrolase, similar to human CD39, which is critical for the intracellular multiplication of *Legionella pneumophila* [41,42]. Recently, a second eukaryotic-type NTPDase, Lpg0971, from *L. pneumophila* was reported, which gives the pathogen the ability to hydrolyze ATP within an intracellular compartment [43]. By contrast, in certain situations, CD39 and CD73 can also control infections and associated inflammation and mortality [44,45]. This evidence suggests that pathogens can also manipulate the host’s adenosine signaling pathways for its own benefit as a strategy to subvert the immune system.

## 3. Adenosine Generated by CD39/CD73 Expression and Impairment of Immunity to Infections

The CD39/CD73 axis has gained increasing importance due to its critical role in the control of inflammation *in vivo* [33,44,46,47,48]. Also, the CD39/CD73 pathway is a critical checkpoint, and through its coordinated activity, it regulates the duration of purinergic signaling in response to various extracellular conditions (e.g., cytokines). Although the concept that adenosine, generated through CD73, provides a feedback to control the tissue damage mediated by host immune responses, it is possible that it can also contribute to a detrimental degree of immunosuppression. During infection, adenosine can harm the host as it dampens the protective anti-microbial response, which is mostly designed to fight the invading pathogen during the early stages of infection. At the same time, over-exuberant inflammation during acute infection can be deleterious to the host. The complexity of the outcome of host responses may depend on the type and stages of infection. The role of adenosine in dampening inflammation has been an area of active research; the relevance of the CD39/CD73-axis and adenosine receptor signaling to host defense against infection has received less attention.

### 3.1. CD73-Regulated Immune Response during Helicobacter-Induced Gastritis and Persistent Infection

*Helicobacter pylori* causes a lifelong infection in humans and the infection of C57BL/6 mice with *H. pylori* or *H. felis* results in chronic active gastritis [49]. Typically, T cells in the stomach are biased largely toward the Th1 phenotype, producing more IFN-γ but very little IL-4 [50,51,52]. Regardless of the strong gastric inflammation associated with *Helicobacter* infection, the bacteria persists for life. Inability to clear the infection may lead to compensatory induction of Th cells with regulatory function to protect the gastric mucosa. In fact, several reports suggest that Th cells resembling Treg are present in the gastric mucosa during *H. pylori* infection of humans and mice [53,54,55,56]. Rad *et al.* [57] have proposed that these Treg contribute to pathogen persistence. Treg may produce sufficient levels of IL-10 and transforming growth factor (TGF)-β to attenuate responses rather than to prevent gastritis totally. Deaglio *et al.* [22] recently reported that, in murine Th cells, the expression of CD39 and CD73 by Treg and the presence of A_2A_AR on activated effector Th cells generate immunosuppressive loops, whereby Treg generates adenosine that inhibits the function of effector Th cells. Similarly, Kobie *et al.* [24] showed that murine Treg express CD73 that converts 5'-AMP from extracellular sources into adenosine, which, in turn, suppresses effector Th cells. We also demonstrated that human CD4^+^CD25^+^Foxp3^+^ Treg from peripheral blood or gastric tissue are enriched for the expression of CD39 and CD73, suggesting that such Treg contribute to adenosine synthesis which, in turn, mediates an anti-inflammatory function [58]. We further showed that when CD73-KO mice were infected with *Helicobacter felis*—a model of *H. pylori* infection—they develop a more severe gastritis with increased levels of IL-2, TNF-α, IFN-γ mRNA and impaired Treg function in gastric tissue than wild-type mice, but cleared the infection more efficiently [58]. Similarly, feeding an A_2A_AR agonist to IL-10 deficient mice suffering from gastritis due to *H. pylori* attenuated gastritis and lowered TNF-α and IFN-γ in the gastric mucosa, but led to increased bacterial colonization [32]. These data suggest that when adenosine/CD73 inhibits pro-inflammatory host responses, it may favor persistent infection. The expression of CD39 and CD73 by Th cells and the enrichment of both enzymes in Treg in humans suggest that Th cells contribute to local adenosine accumulation and the control of inflammation. Moreover, diminished generation of adenosine in CD73-KO mice was associated with impaired Treg function, enhanced gastric inflammation, and reduced levels of colonization upon challenge. These observations support the notion that the production of adenosine and its ability to limit inflammation may contribute to the persistence of *Helicobacter* infection [58].

### 3.2. CD73-Regulated Immune Response during Salmonellosis

*Salmonella* infection is a leading cause of bacterial gastroenteritis in humans. *Salmonella* can lead to severe systemic infection and even death in immunocompromised individuals [59]. *Salmonella* infection in HIV patients is particularly of importance, as the host’s suppressed immunity provides an opportunity for a persistent *Salmonella* infection. The hallmark of the immune response to *Salmonella* infection is characterized by an increase in Th1-type response [60,61,62] and innate host responses generated by phagocyte oxidase and inducible nitric oxide synthase (iNOS) [63,64,65,66,67]. Since CD39/CD73 is abundantly expressed in Th cells that help generate adenosine locally, it is likely that the host immune response during *Salmonella* infection can be regulated by CD73/CD39 pathways. We recently reported that *Salmonella* infection in C57BL/6 mice can down-regulate CD73 expression along with CD39, both at the transcriptional and protein levels in the intestine, liver and spleen, including in Th cells [34]. *Salmonella* infection stimulates a general surge of protective pro-inflammatory cytokines like IL17A and IFN-γ [68,69]. Furthermore, we observed that *Salmonella* infection induced endogenous down-regulation of CD73 resulting in the concomitant production of Th1 cytokines [70], suggesting that the host tries to respond to infection by reducing extracellular adenosine generation which allows increased pro-inflammatory cytokine production critical to fighting the infection.

When CD73 expression was pharmacologically inhibited by adenosine 5'-(α,β-methylene) diphosphate (APCP), a selective CD73 enzyme inhibitor, and then challenged with *Salmonella* whole cell lysate, cultured splenocytes produced increased levels of pro-inflammatory cytokines IL-17A and IFN-γ [34]. Again, CD73 KO mice appeared quite healthy but, upon infection with *Salmonella*, increased levels of intracellular pro-inflammatory cytokines, such as IFN-γ and IL17A were produced by splenocytes and CD4 Th cells. Similarly, the liver tissues from these mice showed significantly higher mRNA expression levels for pro-inflammatory cytokines (IFN-γ, TNF-α, and IL-1β) and inducible nitric oxide synthase (iNOS) [34]. Reduced extracellular adenosine or lack of CD73 expression during infection can help to generate protective pro-inflammatory responses through both an early adaptive response and possibly also by the innate host response, resulting in a reduced *Salmonella* burden in the tissue.

Although CD73 is widely the expressed in many cells, including effector T cells, its expression in CD4^+^CD25^h^ Treg cells is of particular importance, as these immunosuppressive T cells are believed to function through the production of adenosine generated by the expression of CD39/CD73 [22,23,24,34,57,58]. In addition, earlier studies have suggested that CD4^+^CD25^+^Foxp3^+^ Treg cells regulate inflammation and contribute to the persistence of *Helicobacter pylori* infections [57,58]. Depletion of CD4^+^CD25^+^ Treg cells enhances effector T cell activation and reduces pathogen burden during *Leishmania major* infection [71]. Similarly, a correlation was observed between the increased persistence of *Plasmodium falciparum* with upregulation of TGF-β1, Foxp3, and CD4^+^CD25^+^ Treg cells during human malaria infection [72]. Indeed, mouse splenocyte-derived CD4^+^CD25^+^ Treg cells expressed the highest level of CD39/CD73 and *Salmonella* infection reduced the percentage of CD39^+^CD73^+^ cells and also CD73. This indicates Treg cells can become less effective and produce less adenosine to allow pro-inflammatory responses [34]. On the other hand, following infection of CD73-KO mice, in the absence of CD73^+^ cells, Treg cells from these mice may not function correctly [34]. A previous report indicated that CD73-KO mouse-derived Treg cell are functionally deficient in regulating T effector cells [58]. Also, the expression of an anti-inflammatory cytokine, IL-10, did not show any significant up-regulation in the spleen of the infected CD73-KO mice compared with infected wild-type mice. Moreover, IL-4, TGF-β1 and IL-13 mRNA responses, which are also involved in regulating anti-inflammatory responses by Treg cells, were significantly reduced in infected CD73-KO mice [34].

CD73 expression can also effect lymphocyte migration in the draining lymph nodes. Earlier studies showed an approximate 1.5-fold increase in the size of draining lymph nodes and 2.5-fold increased rates of L-selectin-dependent lymphocyte migration from the blood through the HEV (high endothelium venule) in the CD73-KO mice compared with wild-type mice 24 h after lipopolysaccharides (LPS) administration [73,74]. These responses may also be important during bacterial infection or toll-like receptor (TLR) engagement, as CD73 can restrict lymphocyte homing at the infection site thus hampering co-operation between innate and adaptive responses and leading to reduced inflammation and increased bacterial burden. So far, no studies have been done to investigate the role of CD73 on lymphocyte migration during infection. These certainly need further investigation.

### 3.3. CD39/CD73 Expression in the Control of Other Infections

CD39 expression in the control of other infections has also been reported. Theatre *et al.* [45] showed that increased CD39 activity in the airway epithelium of transgenic mice overexpressing human CD39 can promote bacteria-induced inflammation. Upon *Pseudomonas aeruginosa* infection of these mice, increased inflammatory cell (macrophage and neutrophil) infiltration of the airway, increased production of IFN-γ and improved bacterial clearance were observed as compared to infected wild type mice. Thus, the breakdown of ATP into adenosine by overexpressed CD39 can contribute to a neutrophil-dependent inflammatory response that could facilitate pathogen clearance [45].

In another study, CD73-deficient mice had significantly more joint swelling after *Borrelia* infection than WT mice, indicating that dysregulation in adenosine generation may play a role in the persistence of bacterial infection and development of arthritis [75]. In a sepsis model, A_2B_AR blockade or A_2B_AR gene-deleted mice are resistant to cecal ligation and puncture (CLP) mortality and showed enhanced bacterial clearance [76]. Additionally, the absence of A_2B_ receptor promoted antimicrobial activity against Gram-negative bacterial pneumonia [77]. In contrast, Hasko *et al.* [44] reported that during polymicrobial sepsis induced by CLP, CD73-KO mice showed higher mortality in comparison with WT mice, which was associated with increased bacterial burden in blood and lavage fluid and elevated inflammatory cytokine and chemokine levels in the blood and peritoneum, suggesting that CD73-derived adenosine can be beneficial in sepsis. Kolachala *et al.* [78] showed that A_2B_AR-KO mice are susceptible to systemic *Salmonella* infection and had attenuated colonic inflammation. Clearly, some of these contrary findings underline the complexity of adenosine function during infection or sepsis. Further, some pathogens acclimate to exploit immunosuppressive pathways in order to increase their own survival, leading to the possibility that adenosine may favor the survival of pathogen if it is present at the right time and context.

Recently, Boer *et al.* [79] demonstrated the expression of CD39 on *Mycobacterium*-activated human CD8^+^ T cells. They reported a functional role for CD39 on human Bacillus Calmette-Guérin (BCG)-activated CD8^+^CD39^+^ Treg cells and showed that CD39 expression marks a CD8^+^ Treg-cell subset which co-expresses LAG-3, CD25, Foxp3, and CCL4, and that CD39 might play a direct role in exerting CD8^+^ Treg-dependent suppression. In this regard, CD8^+^CD39^+^ Treg cells represent a new player in balancing immunity and inflammation and control mycobacteria infection [79]. The role of CD39 or CD73 on CD8^+^ cells is another new area that deserves attention. Recently, Toth *et al.* [80] reported that HIV-infected patients had reduced CD73 expression in CD8^+^ T-cells and observed that CD8^+^CD73^+^ T cells produced more IL-2 upon stimulation, correlating with immune activation and T-cell exhaustion.

## 4. Adenosine Generated by CD39/CD73 Expression in the Innate Host Response to Infection

### 4.1. Role of Adenosine in Macrophage and Dendritic Cell (DC) Function

Host defense responses to infection are principally orchestrated by mononuclear phagocytes. During infection, these immune cells are activated by a wide array of stimuli, including PAMPS (pathogen-associated molecular patterns, e.g. microbial products like DNA, RNA, LPS, *etc.*) and DAMPS (danger-associated molecular patterns; e.g. ATP, mammalian DNA, protein, host derived peptide or non-peptide regulatory factors). Upon activation, macrophages secrete various inflammatory and anti-bacterial mediators, including cytokines, reactive oxygen species (ROS), and nitric oxide (NO) [66,67,81]. Macrophages, function in the innate host defense against *Salmonella* and other intracellular pathogens by efficient phagocytosis of pathogens, and the production of pro-inflammatory cytokines (e.g. IL-1β, TNF-α). It is likely that adenosine produced through the expression of CD39 and CD73 in macrophages can regulate innate response.

A previous report suggests that macrophages express A_2B_AR, and A_2B_AR blockade enhances bacterial phagocytosis [76]. As with Th cells, the expression of CD39 and CD73 in the macrophage are not well studied. Recent reports suggest that murine peritoneal macrophages mainly express CD39 and it has been found that cAMP upregulates CD39 transcription in murine macrophages [82]. The increase of CD39 mRNA expression in the murine macrophage cell line, RAW 264.7, by cAMP is dependent on protein kinase A (PKA), phosphoinositide 3-kinase (PI3K) and extracellular signal-regulated kinase (ERK) [82]. CD39-deficient peritoneal macrophages pre-stimulated with a TLR-4 agonist (LPS) or a TLR-2 agonist (Pam3CSK4), followed by treatment with ATP, released more IL-1β as compared to WT cells [83]. This increase in IL-1β was not found with agonists of TLR-5 (flagellin) or TLR-3 (poly I:C). Thus, CD39 expression can selectively regulate TLR-mediated cytokine production. It was recently shown that CD39-expressing macrophages played a role in modulating the P2X_7_-dependent production of IL-1β [83,84] in the presence of exogenously added ATP. TLR-stimulated macrophages synthesize, release, and hydrolyze ATP via CD39 to regulate their own activation state. Moreover, the loss of CD39 expression on macrophages prevents regulatory macrophage development and leads to lethal inflammatory responses and septic shock in mice [84].

We have recently reported that RAW 264.7 cells express both CD39 and CD73 at the transcriptional level and *Salmonella* LPS or *Salmonella* whole cell lysate (WCL) treatment downregulates CD39/CD73 expression. Further, CD73 inhibition increases iNOS induction and production of IL1-β [85]. This observation supports our earlier report that liver from infected CD73-KO mice had increased iNOS expression suggesting the possible involvement of macrophages in CD73-mediated innate host response [34]. Hasko *et al.* [86] also suggest that engagement of A_2B_AR agonists suppresses NO production in LPS-treated RAW 264.7 macrophage. Therefore, adenosine may regulate NO production, possibly through macrophage function, to influence the innate host response. It would be interesting to examine the role of CD39/CD73/adenosine axis in the generation of NO by macrophages.

Human monocyte-derived DCs (moDCs) express CD39 and CD73 at both the mRNA and functional level [87]. While murine epidermal DCs express CD39 at the mRNA and protein level [88], murine bone-morrow derived DCs (BMDC) constitutively express CD39 but do not express CD73, even after exposure to TLR agonists, and are therefore unable to convert AMP to adenosine [89]. TGF-β induces CD73 expression in DCs, enabling these cells to generate adenosine within an immune-regulatory microenvironment [3,89].

### 4.2. Role of Adenosine in Neutrophil Function

Neutrophils also play a pivotal effector function during innate defense against bacterial infections [90]. A key feature of these inflammatory cells is their ability to sense hostile conditions, rove toward compromised tissues, and trigger a series of antimicrobial effector mechanisms [91]. Neutrophils widely express CD39 [13] and, to some extent CD73 [92], both of which appear to be critical players in the regulation of neutrophil activity by controlling extracellular purinergic gradients [93]. It is not yet clear how neutrophil expression of CD39/CD73 can regulate the outcome of an infection. In particular, inadequate activity of the CD39/CD73 axis has been associated with amplified and uncontrolled activation of neutrophils [94,95], amplified chemotactic functions [96,97], and an increased adhesion to the vascular endothelium [94,98]. Recently, Barletta *et al.* [77,91] reported that neutrophils from A_2B_AR-KO showed six-fold greater bactericidal activities and enhanced production of neutrophil extracellular traps compared to WT mice when incubated with *Klebsiella pneumoniae*, thus promoting host defense against Gram-negative bacterial pneumonia. More research is needed to understand how neutrophil expression of CD39/CD73 can regulate the outcome of an infection.

### 4.3. Role of Adenosine in Natural Killer (NK) Cell Function

NK cells are effector lymphocytes of the innate host response that control several types of tumor and microbial infections by limiting their propagation and subsequent tissue injury [99]. NK cells can produce various cytokines and chemokines, *i.e.*, IFN-γ, TNF-α, granulocyte-macrophage colony stimulating factor (GM-CSF), macrophage inflammation protein (MIP)-1β and RANTES (Regulated on Activation, Normal T cell Expressed and Secreted) in response to infection and play an important role in the control of viral, parasitic and certain intracellular bacterial diseases [99,100]. In resting human NK cells, the expression of CD39 is reported to be very low (~5%) [13]. According to Beldi *et al.* [14], murine NK cells can abundantly express CD39 mRNA, including A_2A_ and A_2B_ receptors, but very little CD73 mRNA, suggesting that extracellular adenosine can be produced during inflammation and modulate immune function. To date, no reports are available exploring the role of CD39/CD73 pathways in NK cells during active infection.

**Figure 1:**
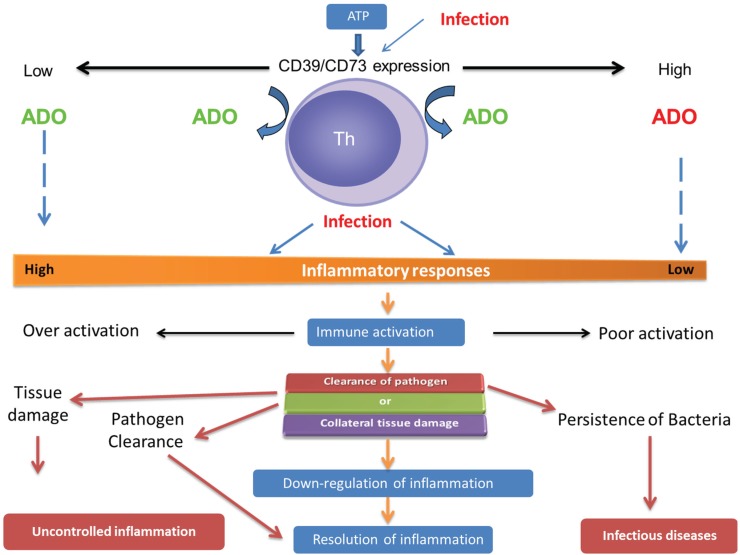
ATP is catabolized by CD39/CD73 expressed in immune cells to generate extracellular adenosine that regulates the outcome of inflammatory response and pathogen persistence during infection. In the normal situation (center), infection-induced immune activation increases the extracellular adenosine level. When immune activation takes place in an adenosine-enriched environment (right side), effector functions of immune cells are insufficient to eliminate pathogens due to poor pro-inflammatory responses; therefore, outgrowth of infectious agents may result. Contrary to this, in the absence or reduced levels of CD39/CD73-Ado (left side), an increased inflammatory response may help the host to clear pathogens. At the same time, uncontrolled activation may lead to collateral tissue destruction. Any unregulated intervention to increase the expression of CD39/CD73 may dampen inflammation and impair immunity to infection.

### 4.4. Role of Adenosine in the Inflammasome

The inflammasome is a multiprotein cytosolic complex expressed by myeloid cells and is part of the innate immune system. The inflammasome is assembled during cellular activation [101,102,103,104]. Purinergic receptors P2X_7_ bind eATP and participate in the activation of inflammasome *via* processing of procaspase-1 to active caspase-1, which, in turn, cleaves pro-IL-1β to mature IL-1β [105,106,107,108]. In macrophages, *Salmonella* also induces the activation of caspase-1, which is necessary for the maturation of the proinflammatory cytokines IL-1β and IL-18. Xiang *et al.* [109] recently reported that ATP helps fight against bacterial infection in mice. ATP induced the secretion of IL-1β and chemokines by murine bone marrow-derived macrophages *in vitro*. Furthermore, the intraperitoneal injection of ATP elevated the levels of IL-1β and chemokines in the mouse peritoneal lavage. A protective role for ATP during bacterial infection was demonstrated and the effect was related to NLRP3 inflammasome activation [109]. Ouyang *et al.* [110] demonstrated that adenosine, acting via the A_2A_ receptor, is a key regulator of inflammasome activity. They further reported that inflammasome regulation by adenosine is initiated by a wide range of PAMPs and DAMPs. A cAMP/PKA/CREB/HIF-1α signaling pathway downstream A_2A_ receptor is activated, which results in up-regulation of pro-IL1β and NLRP3 and greater caspase-1 activation. It is also reported that the inflammasome promotes a pro-inflammatory response which can be dependent on ROS generation [111]. ATP can also drive ROS generation [112] and, particularly, eATP-P2X_7-_mediated cellular ROS generation can also play an important role for microbial persistence and inflammation [113,114]. To our knowledge, there are no published reports assessing whether the CD39/CD73-axis modulates cytokine responses during infection, mediated by NLRs, [101,102,103] which is a topic worthy of future study.

## 5. Conclusions

Adenosine is one of the many biomolecules that accumulate in the inflammatory milieu, conferring pleiotropic effects which can be beneficial or harmful. Extracellular adenosine produced by catabolizing enzymes, like CD39 and CD73, can add to the local adenosine pool. Adenosine generated through CD73 can provide feedback to control the tissue damage mediated by the host immune response. At the same time, during bacterial infection, it can also contribute to a harmful degree of immunosuppression leading to increased pathogen load (Figure 1). The studies outlined in this short review support the notion that adenosine production through the CD39/CD73-axis, and the ability of extracellular adenosine to limit inflammation may favor bacterial survival, thereby broadening the impact of infection.
